# Synaesthetic colours do not camouflage form in visual search

**DOI:** 10.1098/rspb.2007.1457

**Published:** 2008-01-08

**Authors:** C Gheri, S Chopping, M.J Morgan

**Affiliations:** 1 Applied Vision Research Centre, The City UniversityNorthampton Square, London ECV1 0HB, UK; 2 Institute of PsychiatryDe Crespigny Park, Denmark Hill, London SE5 8AF, UK

**Keywords:** synaesthesia, low-level sensory signals, psychophysics, functional magnetic resonance imaging

## Abstract

One of the major issues in synaesthesia research is to identify the level of processing involved in the formation of the subjective colours experienced by synaesthetes: are they perceptual phenomena or are they due to memory and association learning? To address this question, we tested whether the colours reported by a group of grapheme-colour synaesthetes (previously studied in an functional magnetic resonance imaging experiment) influenced them in a visual search task. As well as using a condition where synaesthetic colours should have aided visual search, we introduced a condition where the colours experienced by synaesthetes would be expected to make them worse than controls. We found no evidence for differences between synaesthetes and normal controls, either when colours should have helped them or where they should have hindered. We conclude that the colours reported by our population of synaesthetes are not equivalent to perceptual signals, but arise at a cognitive level where they are unable to affect visual search.

## 1. Introduction

Colour synaesthesia is an intriguing condition in which people have strong associations between visual or auditory stimuli such as numbers, and particular colours. For example, the number ‘2’ (spoken or written) may be experienced as ‘red’. These associations are idiosyncratic, but durable over time (Baron-Cohen *et al*. 1987). A key question for neuroscientists is whether these associations are purely verbal, such as the association that makes us think of ‘Elizabeth’ when we hear or read ‘Queen’, or whether, more interestingly, they evoke experiences like those of real colours.

A recent paper on synaesthesia (Kim *et al*. 2006) states that ‘Based on recent work in a number of laboratories it is *now impossible to dispute* that at least some forms of synaesthesia entail mental experiences that have a genuine perceptual reality’ (added emphasis). The work to which this statement refers purports to show that the perceptual colours experienced by synaesthetes have equivalent effects to real colours in psychophysical tasks. To say that it is ‘impossible to dispute’ this conclusion is a very strong claim, which nevertheless, we shall challenge in this paper. We shall first analyse the existing evidence, and then present an experimental investigation designed to defeat the expectation of synaesthetes that they should show superior performance to normal controls.

A brief introduction may help the non-psychophysical reader to understand the logical issues involved in testing unusual subjects like synaesthetes. It is usual in psychophysics to distinguish between purely phenomenological reports and measures of performance. The reports of synaesthetes that they see certain digits as coloured are purely phenomenological. So is the observation by normal observers that they see a previously ‘yellow’ patch as tinged with red after adapting to a green patch. Phenomenological measures have a long history and an important role to play in the study of perception. However, a potential problem with them is that they are easily influenced by expectations and biases. If we encountered an unusual person who persistently called red stimuli ‘green’, we might wonder whether there was something genuinely unusual about their experience, or whether they were using words in an idiosyncratic way, possibly to attract attention to themselves. We would therefore seek a *performance* measure to supplement the verbal report. For example, a genuinely colour-deficient observer would be expected to be usually poor in identifying the numerals in certain plates of the Ishihara test. If they performed poorly with the plates where we would expect poor performance from the absence of one class of cone receptor, we would be assured that the deficiency was real. If the observer failed on all the plates, we would suspect that they were an impostor. Unfortunately, a sufficiently well-informed malingerer could feign poor performance on certain plates to give the impression of colour deficiency. Performance tests are not infallible, except in the case where the unusual subject performs better than the normal population: superior performance is impossible to feign. If this is not the case, we have to be a little cleverer. One of the Ishihara plates makes use of the phenomenon of ‘colour camouflage’ to engineer a situation where the colour-deficient observer should have superior performance to the normal observer. It is much harder for an observer to feign a condition if they do not know whether they are supposed to be better or worse than the normal observer. This is the logic we shall use in the experiment that we describe below.

The evidence that synaesthetic colours have similar perceptual properties to normal colours can now be assessed. Briefly, we claim that there is no satisfactory evidence that synaesthetic colours produce an objectively measured increase in performance relative to normal controls. In these circumstances, we cannot exclude the possibility that the results given by synaesthetes reflect a natural, probably an unconscious, bias towards behaving as if their synaesthetic colours were real, supported by experimenter expectations (since no blind testing has yet been done, to our knowledge). Crucially, in this literature, far greater effort has been devoted to the collection of data tending to support the reality of synaesthetic colours than to trying to disprove it. In particular, the logical equivalent of the Ishihara test has not been reported until now. The one attempt to turn the synaesthetes' supposed ability against them (§1*a*) only succeeds in showing semantic, not perceptual, interference, as even supporters of the reality of synaesthetic colours allow. The following review of the evidence is not comprehensive, but is intended to illustrate the main kinds of experimental evidence, and their strengths and weaknesses in addressing the question of the reality of synaesthetic colours.

### (a) The Stroop effect

Several studies have shown that synaesthetes are impaired by ‘Stroop's interference’ (Wollen & Ruggiero 1983; Mills *et al*. 1999; Odgaard *et al*. 1999; Dixon *et al*. 2000). In the standard Stroop paradigm, colour names such as RED are printed either in a congruent ink colour (red) or an incongruent colour (green) and subjects are asked to name the colour of the ink. Responses in the incongruent colour are slower than in the congruent. In the ‘synaesthetic Stroop’ paradigm, graphemes are presented in colours that are either congruent or incongruent for each synaesthete. Colour-naming times are slower in the incongruent condition. Because this experiment uses synaesthetes as their own controls, it is not subject to the criticism that they may be more motivated than control subjects, or that they wish to demonstrate that they have special expertise. However, the Stroop experiment does not prove that the colour associations are perceptual. The interference could occur at a semantic level, where two response tendencies, one evoked by the real colour, and the other by the association with the grapheme, collide (MacLeod & Dunbar 1988; MacLeod 1991; Hubbard & Ramachandran 2005). As Palmeri *et al*. (2002) conclude: ‘Indeed, such Stroop's interference could occur even if synaesthetic colours have no perceptual reality whatsoever.’

### (b) The incongruent background effect

Smilek *et al*. (2001) asked a single synaesthetic subject (known by initial ‘C’) and normal subjects to identify a digit presented on a coloured background. If the digit had the same synaesthetic colour as the background (‘congruent’), the synaesthetes were significantly more accurate than if it had a different colour (‘incongruent’). This is a curious result in several ways. First, it is unlikely that the background was exactly the same as the synaesthetic colour, in which case there would have been some residual colour contrast even in the congruent condition. No evidence was presented that *normal* subjects were better at identifying digits at low rather than high chromatic contrast. Second, the result implies that the activation threshold for verbally reporting a digit is higher than for evoking its synaesthetic colour. In other words, the observer has to identify the digit as ‘4’ in order to see it as red, and then seeing it as red slows down the ability to name it as 4 against a red background. Smilek *et al*. suggest a speculative feedback model to explain this paradox, but no demonstration that the model has been instantiated and that it actually works. Finally, and crucially, no evidence is presented that C did significantly better in the ‘non-congruent’ condition than the normal observers; indeed, she did worse than at least one normal observer. This means that there is no evidence for a sensitivity difference, and that C could have produced a congruent versus incongruent difference by unconsciously slowing her response to the congruent stimuli (a bias). In a second task, C localized one of the two possible digits (2 or 4) when presented among 6, 12 or 18 distracter digits (a group of 8s). As in the first task, C was faster on incongruent than on congruent trials. Once again, however, C was no faster on incongruent trials than at least some of the normal controls, leaving open the possibility that the effect is due to a slowing of response on congruent trials. It may also be doubted whether the difference between C and the normal controls reached conventional levels of significance. The difference was significant for C and not for any of the seven normal controls, but it would be necessary to show that this difference is itself significant, by a rank order or similar non-parametric test, and no such test is reported. In a later experiment (Smilek *et al*. 2003), an alphanumeric-colour synaesthete, J, and seven non-synaesthetes searched for target digits presented against backgrounds that were either congruent or incongruent with the colours of J's photisms for the target digits. For J, the slope of the search function for detecting the target digits on incongruent trials was shallower than on congruent trials. In contrast, for the seven non-synaesthetes, the slopes of the search functions for detecting the target digits on congruent and incongruent trials were equivalent. Again, this result could have arisen from a proportional slowing of response on congruent trials.

When the performance measure in the incongruent background effect is the identification of a digit, there is also a possibility that the locus of interference is linguistic, as in the Stroop task. Shown a digit, a synaesthete may have two competing tendencies, for example, ‘two’ and ‘green’. If the background also evokes the name green, it might reinforce the colour-naming tendency over the digit-naming tendency. This explanation may seem far-fetched, but it is no less likely *a priori* than the idea that a congruent background reduces colour contrast of the digit.

### (c) Visual search

If normal subjects try to find a 2 among a set of ‘distracter’ digits such as 5s, their performance deteriorates as the number of distracters increases (the set-size effect). However, if the target is uniquely coloured it ‘pops out’ and performance shows little change with distracter number (Treisman & Gelade 1980). Visual search with both real and synaesthetic colours was investigated by Palmeri *et al*. (2002) in a synaesthete WO, along with normal controls. WO responded more quickly than the mean of the controls, when the search item had a unique synaesthetic colour. WO was not significantly better than controls when target and distracters were a similar (bluish) synaesthetic colour, which is good evidence against a motivational explanation, provided that the different shapes were randomly interleaved rather than blocked. WO showed a clear set-size effect even with synaesthetic colours, leading Palmeri *et al*. to conclude that ‘The pop-out effect reported by WO may not be exactly analogous to the pop-out experienced with real colour’. In contrast, Laeng *et al*. (2004) reported a single case study where the synaesthete PM showed virtually no set-size effect for 2 among 5s when the target had a unique synaesthetic colour. However, further analysis showed that fast response times occurred only when the target was within a few degrees of visual fixation, and the authors conclude that ‘PM's synaesthesia does not occur pre-attentively, but rather is within the focus of attention’.

Edquist *et al*. (2006) presented data from 14 grapheme-colour synaesthetes and 14 matched non-synaesthetic controls, each of whom performed a visual search task in which a target digit was distinguished from surrounding distracters either by its unique synaesthetic colour or by its unique display colour. Participants searched displays of 8, 16 or 24 items for a specific target. In the chromatic condition, target and distracter digits were presented in different colours (e.g. a yellow 2 among blue ‘5s’). In the achromatic condition, all digits in the display were black, but targets elicited a different synaesthetic colour from that induced by the distracters. Both synaesthetes and controls showed the expected efficient (pop-out) search slopes when the target was defined by a unique display colour. In contrast, search slopes for both groups were equally inefficient when the target and distracters were achromatic, despite eliciting distinct colours for the synaesthetes under normal viewing conditions. Edquist *et al*. conclude that, at least for the majority of individuals, synaesthetic colours do not arise early enough in visual processing to guide or attract focal attention.

Sagiv *et al*. (2006) tested the reality of synaesthetic colours in two synaesthetes who perceived greyscale letters and digits in colour. They found no evidence for pre-attentive binding using a visual search paradigm in which the target was a synaesthetic inducer. In another experiment involving colour judgments, they showed that the congruency of target colour and the synaesthetic colour of irrelevant digits modulates performance more when the digits are included within the attended region of space. Sagiv *et al*. propose that the mechanisms giving rise to this type of synaesthesia appear to follow at least some principles of normal binding, and that synaesthetic binding seems to require attention. Their suggestion agrees with the finding (Laeng *et al*. 2004) that synaesthetic facilitation of search occurs only when the target is within a few degrees of fixation.

Studies of visual search, then, have failed in general to find evidence for a pre-attentive effect of synaesthetic colours. There is no convincing evidence from this source that synaesthetic colours are perceptually equivalent to real colours. No studies so far have attempted to slow up visual search by synaesthetic colours using the principle of colour camouflage (Morgan *et al*. 1992).

### (d) Visual grouping

A set of black 2s forming a global shape such as a triangle does not, for normal subjects, stand out from a background of 5s (Beck 1982). However, if the 2s are red and the 5s are green, the global shape is seen much more easily. Ramachandran & Hubbard (2001) investigated whether this was true for synaesthetic colours as well. They reported that two synaesthetes were significantly faster than 40 control subjects at reporting the shape formed by achromatic graphemes that had a different synaesthetic colour from the background graphemes. The experiment was later extended to six synaesthetes (Hubbard *et al*. 2005), five of whom showed superior performance to their control groups (*n*=20). An overall analysis of variance yielded a significant effect of group (synaesthetes versus controls). However, it is interesting to note that one of the control groups was clearly significantly superior to the other five, suggesting some caution in generalizing to the population. If an arbitrarily chosen sample of 20 subjects can be significantly different from 100 other subjects in this task, then the chances of getting six synaesthetes that are different purely by chance must also be considered.

These two studies appear to offer convincing evidence that synaesthetes can do better than controls in a visual grouping task. But does this necessarily mean that they used their synaesthetic colours, rather than that they were trying harder and were more motivated? Clearly, the use of appropriately matched controls is vital in this context. In the experiment by Hubbard *et al*. (2005), the controls were undergraduate students carrying out the experiment for a course credit. It may be doubted whether they were giving the task as much attention as a group of synaesthetes keen to demonstrate their special gift. Another problem is the possibility of perceptual learning. Although the discrimination of graphemes in textures is difficult, it may well improve with practice, as do other kinds of texture segregation (Casco *et al*. 2004). The synaesthetes, if they were giving the task greater attention than the controls, might have been able to speed up their performance by learning. It would be interesting to contrast their performance on early and later blocks of the eight-block (×32 trials) experiment.

A final question is whether the superior performance of the synaesthetes, if it is not due to a motivational factor, necessarily implicates a pre-attentional effect of synaesthetic colour. The display was brief (1 s) but possibly long enough to find several graphemes comprising the shape in a serial search. If this is so, the colours helping the synaesthetes to bind the graphemes into a shape may have been post-attentional. This possibility could have been tested by varying the number of distracters in the task, but this was not done. As in the case of visual search, therefore, it is possible that the shape did not truly pop-out for the synaesthetes.

### (e) Apparent motion

Kim *et al*. (2006) reported results of an investigation in which real colours biased the direction in which observers saw apparent motion in a two-frame movie sequence. In two synaesthetic subjects (WO and LR), their synaesthetic colours also biased perceived motion direction. Kim *et al*. conclude that synaesthetic colours are present at a stage of processing before the matching of tokens for movement perception, but this conclusion is not forced by the data. In fact, since what was measured was a bias, the effect could have arisen at any stage up to the final decision of which button to press. In a two-button forced-choice task, a subject has only to adopt the rule ‘if in doubt press the left-hand button’ to produce a shift in the psychometric function that will emerge as a bias. The fact that there may have been some complicated strategies is indicated by WO's performance when real colours were present in the task. In contrast to normal subjects, WO showed no effect of colour on matching, a fact which the authors were unable to explain. It would be informative to carry out an experiment on a normal subject who was rewarded by a points system for replicating WO's bias. Such experiments are standard in signal detection theory (Green & Swets 1966).

### (f ) Crowding

Ramachandran & Hubbard (2001) reported that synaesthetic colours could also reduce ‘crowding’. Graphemes presented in the periphery of vision are harder to identify it they are surrounded by other flanking graphemes. Two synaesthetes were better than controls at identifying the target grapheme when it had a different synaesthetic colour. Subsequent research with six synaesthetes found the superior-to-normal effect in three of the six (Hubbard *et al*. 2005). This is an extraordinary result, since it seems to imply that graphemes have already been identified before the site of crowding. If this is the case, why is there crowding for achromatic graphemes? The same objection does not apply to studies where crowding is relieved (to a small extent, it should be said) by real colours (Kooi *et al*. 1994; Gheri *et al*. in press) because in that case, colour could increase target salience without prior target recognition. The crowding result is so counterintuitive and hard to explain, that it could serve as a useful focus for replication. If it cannot be replicated, the case would be strengthened that the synaesthetes in the Ramachandran & Hubbard study were somehow responding to their own and the experimenters' expectations.

#### (i) Conclusions from the evidence

The evidence for a low-level colour input from graphemes in synaesthesia is, we suggest, suggestive but far from compelling. The field has suffered from lack of a rigorous effort to rule out experimenter expectations and observer bias as explanations for the data. Apart from the Stroop test, which does not speak to the issue of the perceptual reality of synaesthetic colours, there has been no determined effort to design a task where synaesthetes should be at a disadvantage relative to normal controls. In an attempt to do this, we designed a task in which the presence of colour has been shown to interfere with visual search (Callaghan 1984; Morgan *et al*. 1992). In this task, search for a unique shape is impeded by randomly colouring the texture elements. This is not owing to luminance differences between colours since the colour variation has no detrimental effect for dichromats (Morgan *et al*. 1992); rather it seems that colour is such a strong cue for textural segmentation that it impedes organization based on other attributes, such as shape. We therefore expected to find that synaesthetes would be impaired in a search task by both real and synaesthetic colours. Normal subjects, not having the apparent colours, should be in the same position that dichromats were in the Morgan *et al*. study.

The synaesthetes were a subgroup of the subjects previously tested in an functional magnetic resonance imaging (fMRI) study by Nunn *et al*. (2002) and characterized as having grapheme-colour synaesthesia on the basis of a standard test (Baron-Cohen *et al*. 1987). We tested controls versus synaesthetes with achromatic numerals that had previously been shown to have consistent subjective colours for the synaesthetes, to determine whether their subjective colours would interfere with visual search.

## 2. Material and methods

### (a) Measuring and verifying the subjective colours of synaesthetes

During the course of a previous investigation (Nunn *et al*. 2002), our synaesthetic subjects had used swatches of paint to reproduce as exactly as possible the hues of each of the numbers 0–9. The key to our methodology is that some numbers had the same perceived hue associated with them, while others were different. It was thus important to determine that the hue associations were stable over time. We therefore scanned the paint swatches previously produced by the synaesthetes and used Adobe Photoshop to determine their RGB values on a VGA flat screen. We then presented each subject with a matrix of swatches having the full gamut of RGB values associated with the numbers 0–9 and asked them anew to indicate the number that they associated with each colour. Their choices reproduced their previous associations without error, and furthermore, all subjects agreed that the colours on the screen were a good match to their colour associations.

It should be noted at this point that we never presented these colours in the experiment, and we do not claim that these colours were exact matches to the subjective colours. In the experiment, all the numbers were achromatic (black). The purpose of verifying the colour associations was to show that they were stable, and to be able to present number pairs (e.g. 2 and 8) which had the same subjective colour for a particular subject. The subject's task was to search for a unique item in the array of numbers. A pilot experiment showed that normal observers were faster to find a line of a unique orientation among distracters when it also had a unique (real) colour, and also that they were impaired by colour camouflage in the task.

### (b) Stimuli

**Figure 1:**
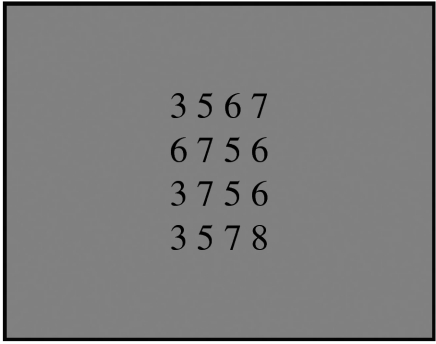
Example of the stimulus array. In this test, all the numbers of the matrix were black (0.8°×0.5°; total stimulus size 4.5°×4.8°; spacing between numbers 1°). The task was indicating the location of the unique number. Two conditions were programmed depending on which colour subjects would associate to each number: the *unique* one, where the target was the only item with a certain colour and the *non-unique* condition where the perceived target colour was repeated on different numbers.

The stimulus array was a 4×4 matrix of different numerals (figure 1) presented in the centre on the screen. The numbers presented were all black and they were all repeated at least once except for the target. (e.g. in the set 3 6 6 7 6 7 5 6 3 7 5 6 3 5 7 8, the number 8 is the target). Two conditions were contrasted for synaesthetes. In the first (the ‘unique condition’) condition, the numbers for each synaesthetic subject were chosen such that the target, as well as being a unique number, also had a unique subjective colour for that subject. In other words, all the distracters had the same subjective colour for that subject, which was different from the colour of the target. In the second condition (‘non-unique’), the target shared its subjective colour with that of at least one of the distracters. Every synaesthete was paired with an age-matched control who was presented with exactly the same stimuli, in the same order. The unique and non-unique conditions were randomly interleaved, and 60 trials were collected in each condition. Constraints on the availability of differently coloured numbers for the synaesthetes meant that the unique condition could contain only five different numbers, while the non-unique contained six different numbers.

### (c) Subjects

The seven synaesthetic subjects were all female, and had previously been subjects in the fMRI study by Nunn *et al*. (2002), during which they were screened for the stability of their grapheme-colour associations. The controls in the present study were age-matched to the synaesthetes (synaesthetes: mean 45.7 years, range 30–56; controls: mean 45.5 years, range 29–55). All subjects were tested with the Farnsworth D15 to check for any abnormal colour vision and verify their colour discrimination ability. None of the subject showed any colour vision deficiency on this test.

## 3. Results

To obtain a normal distribution of data and to be able to use parametric statistics, all the reaction time data were logarithmically transformed.

**Figure 2:**
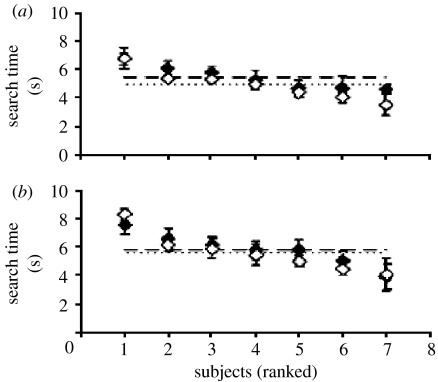
The graphs show the mean reaction time for each subject in the two conditions. (*a*) Unique and (*b*) non-unique conditions. The two lines represent the means of the two groups: the black dashed line for synaesthetes (filled diamonds) and the dotted line (open diamonds) for controls. There was no significant difference between synaesthetes and controls.

Per cent correct was high for all subjects in both conditions and did not differ significantly between conditions. Reaction time data are shown in figure 2. Our predictions were that synaesthetes would be faster than controls in the unique condition, but slower in the non-unique one. However, as is evident from inspection, there was no significant difference between groups in either the two conditions. (*F*=4.25, *p*=0.87), and there were no significant interactions (*F*=4.25, *p*=0.56) between groups and conditions.

A further hypothesis was that synaesthetes would be better in the unique condition compared with the non-unique one, but there was no significant difference (*F*=4.25, *p*=.11). Individual *t*-tests showed a significant difference between conditions in two of the seven synaesthetes, but this was also found in five of the controls. The probable explanation of this difference is that a smaller range of numbers was used in the unique versus the non-unique condition, as explained in §2.

## 4. Conclusion

It should be noted that we do not claim that our task involved pre-attentive search. The subjects probably had to scan the array serially to determine which number was unique. Given a growing consensus (see §1) that synaesthetic colours arise, at least in many cases, post-attentively, this should have helped the synaesthetes to use their subjective colours. However, our data have failed to confirm the prediction that synaesthetes will be impaired by synaesthetic colours in a visual search task where real colours impair performance. They therefore do not support the proposition that synaesthetic colours, at least in our subjects and with our experimental design, are ‘perceptual’ rather than ‘conceptual’. The possibility remains that synaesthetic colours are perceptual but too weak to impair performance like wavelength-based colours. Against this, strong effects of synaesthetic colour have been reported in previous experiments. In the Kim *et al*. (2006) study of apparent motion, the effect of synaesthetic colour was equivalent to real colour in one subject, and stronger in the other.

On the other hand, our results are concordant with others suggesting that synaesthesia may result from the activity of areas concerned with language (Simner 2007) and visual feature segregation or that colours perceived might arise from frontal brain areas that lie beyond the perceptual processing hierarchy (Paulesu 1995; Rich & Mattingley 2002).

An important question is whether synaesthetes are a homogeneous group. Some investigators have posited two subgroups: ‘higher synaesthetes’, in whom the cross activation occurs between ‘higher colour areas’; and ‘lower synaesthetes’ where quite early visual areas are activated (Ramachandran & Hubbard 2003; Hubbard *et al*. 2005). It has also been suggested that only some kinds of synaesthetes, called ‘projectors’, actually see their synaesthetic colours projected into the outside world (Smilek *et al*. 2001). More recently, Ward *et al*. (2006) compared the behavioural performance of seven projector and seven associator synaesthetes, and showed that that this distinction does not map on to the higher–lower distinction. They replicated previous research showing that projectors are faster at naming their synaesthetic colours than veridical colours, and that associators show the reverse profile. Synaesthetes who project colours into external space but not on to the surface of the grapheme behave like associators on this task. In a second task, graphemes presented briefly in the periphery were more likely to elicit reports of colour in projectors than associators, but the colours tended to be accurate only when the grapheme itself is also accurately identified. Ward *et al*. propose an alternative model of individual differences in grapheme-colour synaesthesia that emphasizes the role of different spatial reference frames in synaesthetic perception.

We do not know into which category our synaesthetes fell. We concede the possibility that our samples were of the kind, for whom subjective colour is not an aid to visual search. On the other hand, our synaesthetic subjects had been previously characterized in the fMRI study by Nunn *et al*. (2002) as showing activation of V4/V8 by spoken words having stable colour associations, and this would seem to indicate a relatively low-level colour signal. Either the V4/V8 signal is not sufficient to help with visual search; or our subjects have V4/V8 activation to spoken but not visual input. Our result taken with that of Nunn *et al*. (2002) has the interesting implication that V4/V8 activation is not necessarily associated with the perceptual experience of colour.
